# Characteristics of T-lymphocyte subsets in patients with severe fever with thrombocytopenia syndrome complicated with invasive pulmonary aspergillosis: a retrospective study

**DOI:** 10.3389/fimmu.2025.1748830

**Published:** 2026-01-23

**Authors:** Ying Xu, Yang Liu, Yajun Qian, Ning Liu, Jian Tang, Danjiang Dong, Mao Xia, Weiwei Wu, Qin Gu

**Affiliations:** 1 Department of Intensive Care Medicine, Nanjing Drum Tower Hospital, Affiliated Hospital of Medical School, Nanjing University, Nanjing, China; 2 China Hospital Reform and Development Research Institute of Nanjing University, Nanjing Drum Tower Hospital, Nanjing, China; 3 Department of the Laboratory, Nanjing Drum Tower Hospital, Affiliated Hospital of Medical School, Nanjing University, Nanjing, China; 4 Department of Medicine, Dinfectome Inc., Nanjing, Jiangsu, China

**Keywords:** aspergillosis, immunity, severe fever with thrombocytopenia syndrome, T-lymphocyte subsets, transcriptome analysis (RNA-seq)

## Abstract

**Objectives:**

Immunosuppressed patients often acquire invasive pulmonary aspergillosis (IPA). In recent years, the incidence of patients with severe fever with thrombocytopenia syndrome (SFTS) complicated with IPA has increased. This study aimed to investigate the characteristics of the counts of T-lymphocyte subsets in patients with SFTS combined with IPA and explore their predictive value for IPA infection in SFTS patients.

**Methods:**

We conducted a retrospective review of all patients with SFTS admitted to Nanjing Drum Tower Hospital between January 2016 and August 2022. The patients were divided into IPA and the non-IPA group. Clinical symptoms, laboratory findings, comorbidities, and overall prognosis were collected. Transcriptome sequencing was performed on six of the samples.

**Results:**

A total of 99 SFTS patients were included, of whom 21 (21.2%) developed IPA. The 28-day mortality rate (33.3%) was higher in the IPA group than in the non-IPA group. The IPA group had a significant decrease in the absolute counts of total lymphocytes and CD4+ T lymphocytes and an increase in the ratio of CD4/CD8 T lymphocytes. ROC curves showed that the sensitivity and specificity for predicting the occurrence of IPA in SFTS patients was 50% and 95%, respectively, when the cut-off value for CD4+ T lymphocytes was 386 cells/μL. The transcriptome analysis revealed significant differences in host gene expression profiles between IPA and non-IPA groups, with notable enrichment in KEGG pathways related to metabolism, cellular functions, and systemic processes.

**Conclusions:**

Patients with an absolute count of CD4+ lymphocytes below 386 cells/μL are at risk of acquiring secondary IPA. It is necessary to screen the counts of T-lymphocyte subsets in SFTS patients after admission.

## Introduction

Severe fever with thrombocytopenia syndrome (SFTS) is an emerging infectious disease first confirmed in Chinese patients in 2011 (1). Subsequently, Japan, South Korea, Myanmar, Pakistan, and other Southeast Asian countries have reported cases, but most cases are still occurring in China (2–6).

A member of the Phlebovirus genus, the SFTS virus (SFTSV) is a negative single-stranded RNA virus. Ticks are a known vector of SFTS because the nucleic acid sequence of SFTSV recovered from ticks exhibits 95% similarity with that obtained from certain SFTS patients who had a documented history of tick bites (1). Studies have also confirmed that SFTSV can be transmitted from person to person through patient blood, especially when the viral load in the blood is high (7).

SFTS has apparent regional clustering; most patients are farmers living in hilly and mountainous areas or working in forests. The incidence of SFTS also has pronounced seasonality, generally starting in March, peaking from May to July, and ending in November (8). The incubation period of SFTS ranges from 5 to 14 days and is affected by viral load or infection route (9). The clinical manifestations of SFTS are nonspecific, including fever, leukopenia, thrombocytopenia, gastrointestinal symptoms, fatigue, conjunctival congestion, abdominal pain, diarrhea, hematuria, and lymphadenopathy. Patients with severe disease progress rapidly and die of multiple organ failure (MOF), including acute kidney injury, myocarditis, arrhythmia, and meningoencephalitis (9). The mortality rate of SFTS has been reported to be 6%-21%. Adverse prognostic factors include advanced age, altered mental status, elevated serum LDH and AST levels, prolonged APTT, and high serum viral RNA load (10, 11).

In recent years, an increasing number of studies have reported a high proportion of secondary invasive aspergillosis (IPA) in SFTS patients, ranging from 32% to 56%, and a high mortality rate of 37.9%, which is much higher than that of patients without IPA infection (12, 13). IPA is most common in immunosuppressed or immunodeficient patients, such as those with organ transplantation, immune system diseases, hematological diseases, and long-term use of hormones and immunosuppressive agents. However, prior to infection, most SFTS patients have been reported to be healthy with no immunosuppression history. Therefore, we reviewed T-lymphocyte subsets in SFTS patients with IPA and analyzed their value in the early warning prediction of IPA and in prognosis.

## Methods

### Study population

A retrospective analysis was carried out on patients who were hospitalized to Nanjing Drum Tower Hospital, a tertiary hospital located in Nanjing, China, between January 2016 and August 2022 and had confirmed SFTS. The Ethics Committee at Drum Tower Hospital, which is connected to Nanjing University’s Medical School, gave its approval for this study (No. 2021-522-02). To reduce potential confounding from pre-existing immunosuppression, we reviewed baseline comorbidities and medication history at admission. Patients with documented immunocompromising conditions or treatments (e.g., HIV infection, hematologic malignancy, solid organ/hematopoietic stem cell transplantation, chemotherapy, or long-term systemic corticosteroid or other immunosuppressive therapy) were identified and excluded if present in the records. Due to the retrospective nature of the investigation, individual patient informed permission was not required.

### Diagnostic criteria for SFTS and IPA

The SFTS diagnosis was confirmed by the detection of SFTSV RNA using polymerase chain reaction (PCR) with the DiaStar 2X OneStep RT–PCR Pre-Mix kit (SolGent, Daejeon, South Korea) as previously described (14). The National Institute of Allergy and Infectious Diseases Mycoses Study Group (EORTC/MSG) and the European Organization for Research and Treatment of Cancer/Invasive Fungal Infections Cooperative Group recommendations were used to diagnose IPA (15). The guidelines classify IPA diagnosis into proven, probable, and suspected IPA. The diagnosis of proven IPA was made on the basis of microscopic evidence of mycelial development and a positive *Aspergillus* spp. culture from an endobronchial biopsy rather than bronchoalveolar lavage fluid (BAL), regardless of the host variables or clinical symptoms. Based on the host variables, clinical characteristics, and mycological evidence of aspergillosis, a probable IPA diagnosis was made. The three types of CT signs—solid, well-defined nodules with or without the halo sign, the air crescent sign, or the presence of cavities—that were seen on at least one chest CT scan were among the clinical characteristics. Mycological proof was defined as either a positive culture, microscopic evidence obtained with qualified lower respiratory tract Aspergillus specimens, or a galactomannan (GM) optical index >0.5 from a blood sample or BAL sample. Only individuals with a confirmed or suspected diagnosis of IPA were included in this study.

### Data collection

Data was gathered by investigators from the Nanjing Drum Tower Hospital’s computerized medical records system, which is connected to Nanjing University’s Medical School in Nanjing, Jiangsu, China. T-lymphocyte subsets, comorbidities, test results, clinical symptoms, demographic information, and overall prognosis were evaluated.

### Testing methods for T-lymphocyte subsets

T-lymphocyte subsets were counted with a FACS Calibur flow cytometry analyzer produced by BD company (BD MultTEST IMK Kit) at our hospital. The main antibodies included CD3-FITC/CD88-PE/CD45-PreCP/CD4-APC and CD3-FITC/CD16 + 56-PE/CD45-PreCP/CD19-APC. Lymphocytes were gated using CD45/SSC and FSC/SSC to exclude debris, then T cells (CD3+) were subdivided into CD4+ and CD8+ subsets, with B cells and NK cells defined as CD3−/CD19+ and CD3−/CD16 + 56+, respectively. Instrument settings were adjusted using an isotype control (IgG/IgG2a), and consistent gating was applied across samples; absolute counts (cells/µL) were obtained using CellQuest.

The corresponding fluorescent antibody was added to the Falcon tube, and then 2 ml of anti-coagulation peripheral blood was added, vortexed, shaken for a few seconds, and placed in the dark at room temperature for 20 minutes. Then, 2 ml of 1 × hemolysin was added and shaken for a few seconds, incubated in the dark at room temperature for 15 min, and centrifuged at 1000 r/min at room temperature for 5 min. After discarding the supernatant, 2 ml of PBS was added and vortexed for several seconds. After centrifugation at 1000 r/min for 5 min at room temperature, 0. 5 mL of PBS was added to the supernatant and then used for detection. The absolute number of positive cells in the sample (cells/μL) was obtained by flow cytometry software (cell quest). The negative control tube (IgG/IgG2a) was used to adjust the FSC Amplifil and SSC Detector so that the three groups of cells appeared uniformly on the FSC-SSC dot plot. The logical gate method (CD45-SSC gate and FSC-SSC gate) was used to reduce the interference of impurities.

### RNA-seq

RNA analysis was performed on blood samples from six SFTF patients, including three patients with IPA and three patients without IPA. RNA was extract with the QIAamp Viral RNA Mini Kit (Qiagen). For RNA library construction, the rRNA was removed from the total RNA, and the library was constructed after purification. The Agilent 2100 Bioanalyzer system (Agilent, Santa Clara, CA, USA) was employed for quality control and DNA libraries were 75bp single-end sequenced on Illumina NextSeq 550Dx (Illumina, San Diego, CA, USA).

Base calling was performed on bcl2fastq v2.20 (Illumina, Inc.) to generate sequence reads in FASTQ format (Illumina 1.8+ encoding). Quality control (QC) was performed with Trimmomatic (version 0.36). STAR (version 2.5.3a) is used for transcriptome mapping followed by isoform and gene level quantification performed by RSEM (version 1.2.31). Differential expression analysis was conducted by R packages DESeq2 (version 1.22.2). Differentially expressed genes were selected by Fold Change > 2 and *p*-value < 0.05. Corresponding volcano plots and heatmaps were generated by in-house R scripts. The Gene Ontology (GO) and the Kyoto encyclopedia of genes and genomes (KEGG) enrichment analysis were performed by KOBAS (version 3.0).

The CIBERSORTx algorithm was employed to deconvolute and analyze immune cell composition. Gene expression data in FPKM (Fragments Per Kilobase Million) format were used, comprising transcriptomic profiles from three SFTF patients with IPA and three SFTS patients without IPA. Analysis was performed using the standard LM22 signature gene matrix via the CIBERSORTx platform. Key parameters were set as follows: the number of permutations was set to 1000 to enhance the robustness of statistical inference, and quantile normalization was disabled (QN=F), as recommended for pre-normalized data formats like FPKM in relative mode. The algorithm estimated the relative proportions of 22 immune cell subsets, including: naive and memory B cells, plasma cells, CD8+ T cells, naive CD4+ T cells, memory CD4+ T cells, follicular helper T cells, regulatory T cells, gamma delta T cells, natural killer cells, monocytes, M0, M1, and M2 macrophages, resting and activated dendritic cells, resting and activated mast cells, eosinophils, and neutrophils. Differences in immune cell proportions between sample groups were assessed using the Wilcoxon rank-sum test.

### Statistical analysis

The statistical software SPSS 23.0 (SPSS Inc., Chicago, USA) was used for all analyses. Numerical or categorical variables are used to report data. The continuous, normally distributed variables were compared using ANOVA, and the rates were compared using the chi-square test. The rank sum test was used to analyze nonnormally distributed data, which are represented as medians (*p* 25, *p* 75). ROC curve analysis was used to assess the T-lymphocyte subsets’ sensitivity and specificity in IPA prediction. Optimal cut-off values were determined using the Youden index (maximum sensitivity + specificity - 1). The univariate and multivariate logistic regression analyses were conducted to identify the risk factors associated with the development of IPA in patients with SFTS. To reduce the risk of overfitting, the number of predictors included in the multivariable logistic regression was deliberately restricted. Candidate variables were evaluated based on univariate association and clinical relevance, and the events-per-variable (EPV) in the final multivariable model was 21. The outcomes are presented as 95% confidence intervals (CI) corresponding to the adjusted odds ratios (ORs). A significant difference was deemed to exist if the *p*-value was less than 0.05 in all two-sided significance tests.

## Results

### Clinical characteristics

**Figure 1:**
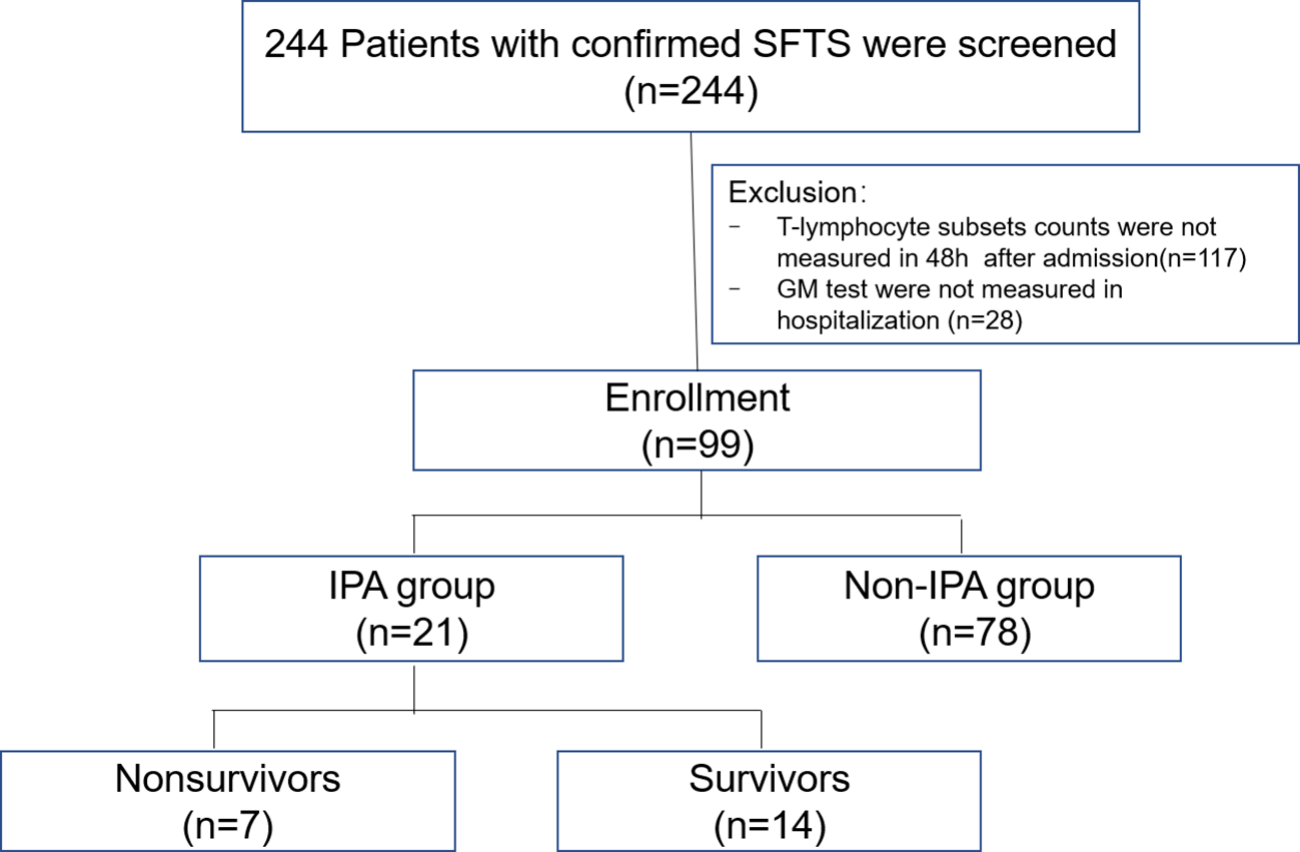
Schematic flow chart of patient enrolment.

Between January 2016 and August 2022, 244 patients with confirmed SFTS were screened. Of them, 117 patients did not have measurements for T-lymphocyte subsets 48 h after admission, and 28 did not have measurements for the GM test; thus, 99 patients were enrolled in this study. Among them, 21 had proven or probable IPA diagnoses and were in the IPA group, and 78 were in the non-IPA group. There were seven in-hospital deaths in the IPA group, which was one-third of the IPA group (**Figure 1**).

The baseline characteristics, clinical symptoms, admission laboratory results, and comorbidities that distinguish IPA patients from non-IPA patients are shown in **Table 1**. The median age of the 51 patients (51.5%) was 62 years old. Of them, 51 were male. The two groups did not differ in terms of age or gender. Eight-five (85.9%) patients had participated in rural activities before the onset of SFTS, and nine (9.1%) patients had a history of tick bites. Ninety-five (95%) patients presented with a high fever, and other symptoms included cough (7.1%), vomiting, abdominal pain and diarrhea (25.3%), headache (12.1%), unconsciousness (19.2%) and bleeding (19.2%). More patients in the IPA group experienced unconsciousness than in the non-IPA group (*p* < 0.05).

**Table 1:** Clinical characteristics of the IPA and non-IPA groups of SFTS patients.

Characteristics	All patients (n = 99)	IPA group (n = 21)	Non-IPA group (n = 78)	*p*-value
Demographics				
Sex, male (%)	51 (51.5)	9 (42.9)	42 (53.8)	0.258
Age	62.68 ± 13.13	64.48 ± 11.09	62.19 ± 13.65	0.482
Residence, rural area (%)	93 (93.9)	19 (90.5)	74 (94.9)	0.375
Days of onset prior to hospitalization	8.31 ± 2.71	7.81 ± 2.20	8.45 ± 2.82	0.339
Exposure to related activities, n (%)				
Rural activities	85 (85.9)	17 (81.0)	68 (87.2)	0.339
History of tick bites	9 (9.1)	2 (9.5)	7 (9.0)	0.610
Clinical manifestations, n (%)				
Fever> 38.5°C	95 (96.0)	20 (95.2)	75 (96.2)	0.621
Cough	7 (7.1)	2 (9.5)	5 (6.4)	0.460
Vomiting, abdominal pain, diarrhea	25 (25.3)	6 (28.6)	19 (24.4)	0.445
Headache	12 (12.1)	5 (23.8)	7 (9.0)	0.076
Unconsciousness	19 (19.2)	8 (38.1)	11 (14.1)	0.019
Hemorrhage	19 (19.2)	5 (23.8)	14 (17.9)	0.372
Laboratory testing, M (Q1, Q3)				
Leukocytes (normal 4-10 × 109/L)	3.10 (1.85, 4.90)	3.20 (2.00, 5.10)	3.05 (1.83, 4.88)	0.87
Platelets (normal 100-300 × 106/L)	44.00 (32.00, 67.75)	39.00 (28.00, 91.00)	48.00 (32.00, 67.00)	0.61
ALT (normal 0–40 U/L)	61.95 (43.28, 110.10)	52.60 (43.28, 73.38)	62.00 (46.50, 119.40)	0.68
AST (normal 0–40 U/L)	172.00 (98.00, 341.50)	181.10 (69.00, 284.00)	164.35 (100.50, 346.75)	0.94
LDH (normal 109–245 U/L)	1604.00 (1041.00, 2150.00)	1605.00 (1056.00, 1841.50)	1531.00 (1051.25, 2424.50)	0.84
BUN (normal 2.9-7.5 mmol/L)	4.48 (3.21, 6.13)	5.75 (3.97, 7.65)	4.20 (3.12, 6.03)	0.37
Scr (normal 44-106 µmol/L)	61.00 (49.00, 75.45)	66.00 (62.00, 88.20)	59.00 (48.00, 71.57)	0.03
Amylase (normal 25–115 U/L)	125.00 (82.50, 232.00)	175.00 (108.00, 492.00)	115.50 (78.50, 220.25)	0.08
Comorbidity, n (%)				
Respiratory failure	11 (11.1)	5 (23.8)	6 (7.7)	0.052
Vasopressor use	17 (17.2)	8 (38.1)	9 (11.5)	0.008
Bacteraemia	6 (6.1)	3 (14.3)	3 (3.8)	0.108
Maximum viral load, log copies/mL, median (IQR)	148 (56-403)	148 (55-403)	148 (54-403)	0.971
Prognosis, n (%)				
28 d-mortality	17 (17.2)	7 (33.3)	10 (12.8)	0.035

Laboratory findings at admission are shown in **Table 1**. The leucocyte, platelet count, serum aspartate aminotransferase (AST), aspartate aminotransferase (ALT), lactic dehydrogenase (LDH) and amylase levels had no dramatic difference between the IPA and non-IPA groups. The level of serum amylase in the IPA group was significantly higher than that in the non-IPA group (*p* < 0.05). Eleven (11%) patients had respiratory failure, seventeen (17.2%) patients used vasopressors, and six (6.1%) patients had bacteraemia. Of the bacteraemia cases, 4 were due to infection with *Staphylococcus aureus*, 1 with *Elizabethkingia*, and 1 with *Klebsiella pneumoniae*. The proportions of patients with vasopressor use and bacteraemia in the IPA group were higher than those in the non-IPA group (*p* < 0.05). There was no significant difference in the maximum viral load between the two groups (*p*>0.05). The 28-day mortality rate of the IPA group (7/21, 33.3%) was significantly higher than that of the non-IPA group (10/78, 12.8%).

SFTS, severe fever with thrombocytopenia syndrome; IPA, invasive pulmonary aspergillosis; AST, aspartate aminotransferase; ALT, alanine aminotransferase; LDH, lactic dehydrogenase; BUN, blood urea nitrogen; Scr, serum creatinine.

SFTS patients with IPA were divided into survivors and nonsurvivors (**Table 2**). The proportions of patients with headache, unconsciousness and vasopressor use were significantly higher among the nonsurvivors than the survivors.

**Table 2:** Clinical characteristics of the survivors and nonsurvivors with SFTS and IPA.

Characteristics	Survivors (n =14)	Nonsurvivors (n = 7)	*p*-value
Demographics			
Sex, male (%)	5 (35.7)	4 (57.1)	0.319
Age	68.14 ± 10.60	57.14 ± 8.51	0.028
Residence, rural area (%)	70 (85.4)	15 (88.2)	0.554
Days of onset prior to hospitalization	7.50 ± 2.18	8.43 ± 2.30	0.376
Exposure to related activities, n (%)			
Rural activities	13 (92.9)	6 (85.7)	0.567
History of tick bites	1 (7.1)	1 (14.3)	0.567
Clinical manifestations, n (%)			
Fever> 38.5°C	13 (92.9)	7(100)	0.667
Cough	1 (7.1)	1 (14.3)	0.567
Vomiting, abdominal pain, diarrhea	5 (35.7)	1 (14.3)	0.314
Headache	0 (0)	5 (71.4)	0.001
Unconsciousness	3 (21.4)	5 (71.4)	0.041
Hemorrhage	4 (28.6)	1 (14.3)	0.443
Laboratory testing, M (Q1, Q3)			
Leukocytes (normal 4–10 × 10^9^/L)	3.50 (2.08, 5.25)	3.20 (1.55, 3.80)	0.502
Platelets (normal 100–300 × 10^9^/L)	39.00 (26.50, 81.75)	48.00 (30.50, 70.50)	0.794
ALT (normal 0–40 U/L)	68.50 (46.80, 75.00)	42.70 (38.20, 58.40)	0.222
AST (normal 0–40 U/L)	195.00 (152.50, 367.50)	117.35 (48.95, 213.28)	0.339
LDH (normal 109–245 U/L)	1768.00 (1243.00, 1878.00)	858.50 (665.25, 1051.75)	0.218
BUN (normal 2.9-7.5 mmol/L)	5.74 (3.14, 5.79)	8.26 (6.43, 12.50)	0.267
Scr (normal 44-106umol/L)	65.50 (62.00, 76.33)	79.00 (62.00, 125.45)	0.351
Amylase (normal 25–115 U/L)	160.50 (111.00, 234.25)	200.00 (131.50, 539.00)	0.526
Comorbidity, n (%)			
Respiratory failure	2 (14.3)	3 (42.9)	0.182
Vasopressor use	3 (21.4)	5 (71.4)	0.041
Bacteraemia	2 (14.3)	1 (14.3)	0.753
Maximum viral load, log copies/mL, median (IQR)	148 (56-403)	212 (15-316)	0.643

### T-lymphocyte subsets in the IPA and non-IPA groups

**Figure 2:**
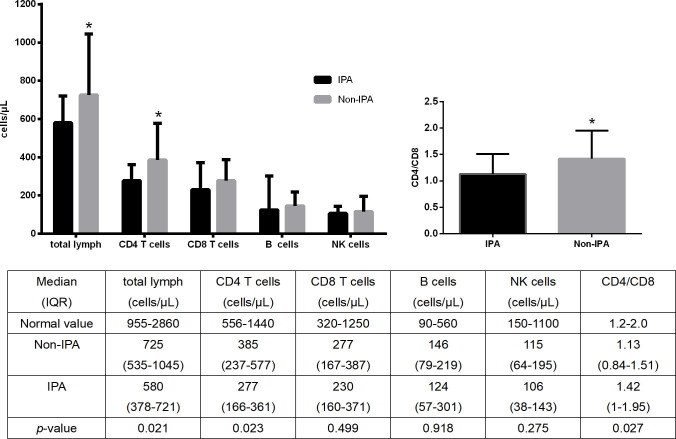
Comparison of T-lymphocyte subset levels between the IPA and non-IPA groups in SFTS patients. *p < 0.05.

**Figure 2** illustrates that the IPA and non-IPA groups had median total lymphocyte counts, as well as CD4+ T-lymphocyte, CD8+ T-lymphocyte, and NK-cell counts, that were significantly below the normal range. The absolute counts of CD4+ T cells and total lymphocytes were significantly lower in the IPA group than in the non-IPA group (*p* < 0.05). There was no discernible difference between the two groups’ total counts of CD8+ T cells, B lymphocytes, or NK lymphocytes. Compared to the non-IPA group, the IPA group’s CD4/CD8 ratio was significantly lower (*p* < 0.05). The representative FACS plots for the IPA and non-IPA groups were presented in **Supplementary Figures 1**, **2** respectively.

**Figure 3:**
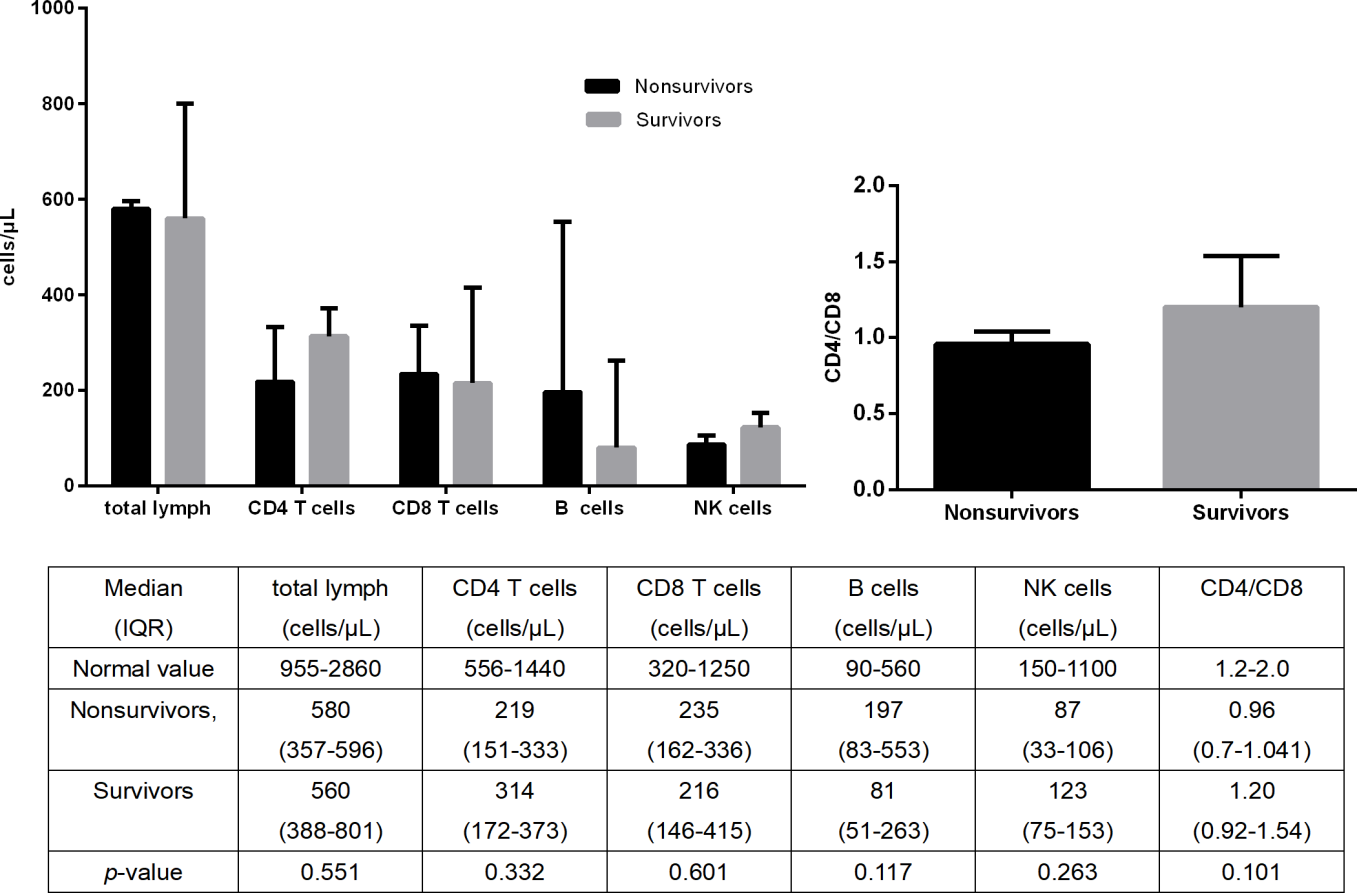
Comparison of T-lymphocyte subset counts between the survivors and nonsurvivors with SFTS combined with IPA.

The counts of T-lymphocyte subsets in survivors and nonsurvivors in the IPA group are shown in **Figure 3**. There was no significant difference in the absolute counts of total lymphocytes, CD4+ T lymphocytes, CD8+ T lymphocytes, B lymphocytes, and NK lymphocytes between the two groups (*p*>0.05).

### ROC curves for identifying IPA risk

**Figure 4:**
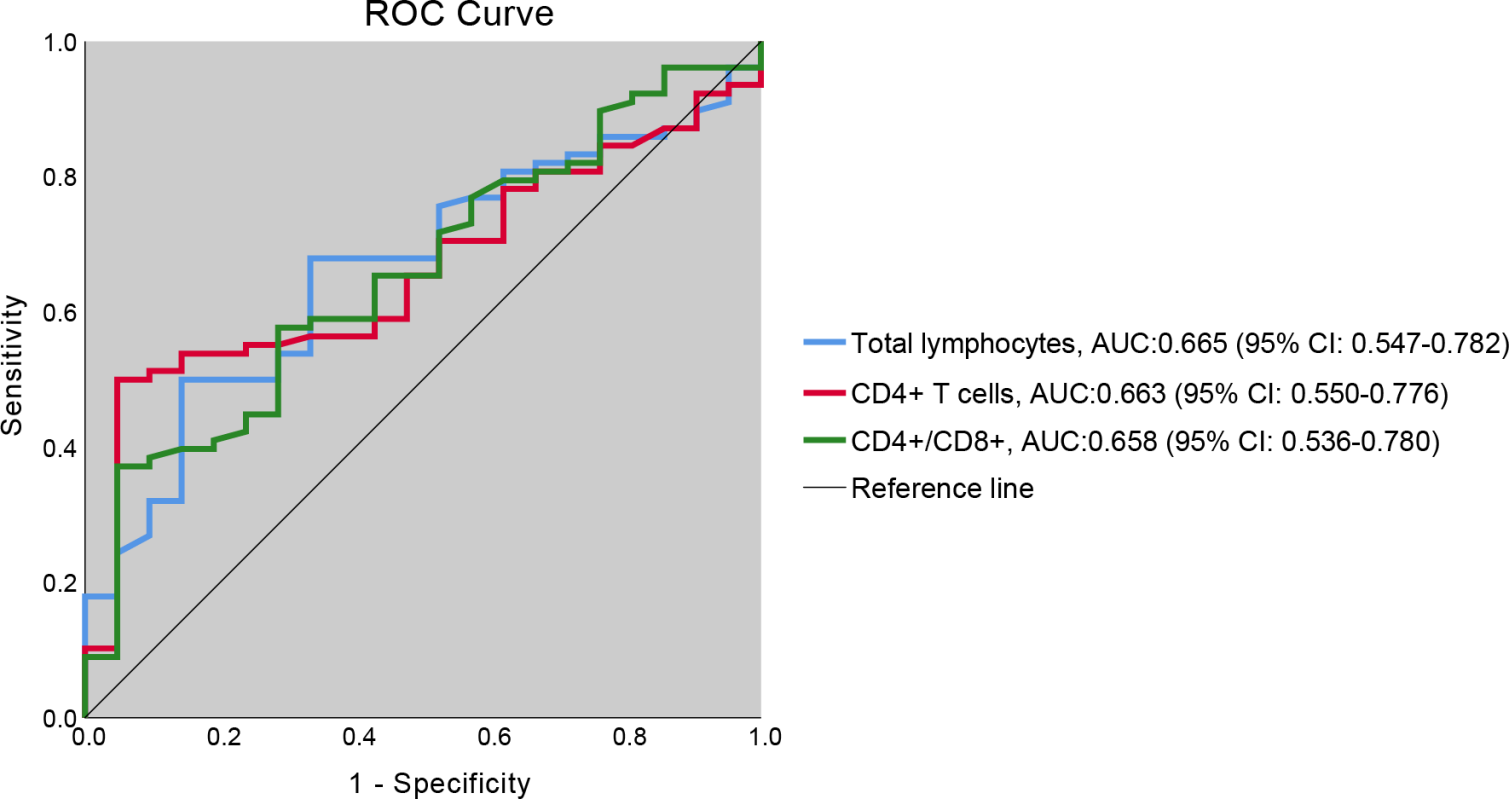
ROC curve analysis of T-lymphocyte subset counts for predicting IPA in SFTS patients.

The sensitivity and specificity of T-lymphocyte subsets for identifying patients at risk of IPA were evaluated using ROC curve analysis (**Figure 4**). The AUCs (95% CI) were 0.665 (95% CI: 0.547-0.782) for total lymphocytes, 0.663 (95% CI: 0.550-0.776) for CD4+ T lymphocytes, and 0.658 (95% CI: 0.536-0.780) for the CD4/CD8 ratio, indicating moderate discriminatory in this cohort. Cut-off values were determined using the Youden index. Using this approach, a CD4+ T-lymphocyte cut-off of 386 cells/µL yielded a sensitivity of 50% and a specificity of 95%, suggesting that a low CD4+ T-cell count may serve as a potential risk indicator for IPA development in SFTS patients.

### Univariate and multivariable regression analyses of risk factors for the development of IPA in SFTS patients

Univariate logistic regression showed that unconsciousness, respiratory failure, vasopressor use, amylase >115 U/L, total lymphocytes < 743 cells/µL, CD4 T cells < 386 cells/µL and CD4/CD8 < 1.68 were associated with for the development of IPA in SFTS patients. Multivariable logistic regression analysis identified CD4 T cells < 386 cells/µL as the only independently associated factor of IPA development in SFTS patients (**Table 3**).

**Table 3:** Univariate and multivariable regression analyses of risk factors for the development of IPA in SFTS patients.

Variable	Univariate		Multivariable	
	OR (95% CI)	*p*-value	OR (95% CI)	*p*-value
Unconsciousness	3.748 (1.264–11.118)	0.017		
Respiratory failure	3.750 (1.017–13.823)	0.047		
Vasopressor use	4.718 (1.537–14.484)	0.007		
Bacteraemia	4.167 (0.776–22.378)	0.096		
Amylase>115U/L	3.284 (1.012–10.662)	0.048		
Total lymph<743 cells/µL	6.000 (1.635–22.023)	0.007		
CD4 T cells<386 cells/µL	20.000 (2.557–156.422)	0.004	13.868 (1.336-143.949)	0.028
CD4/CD8<1.68	11.837 (1.508-92.889)	0.019		

### Results of RNA-seq

**Figure 5:**
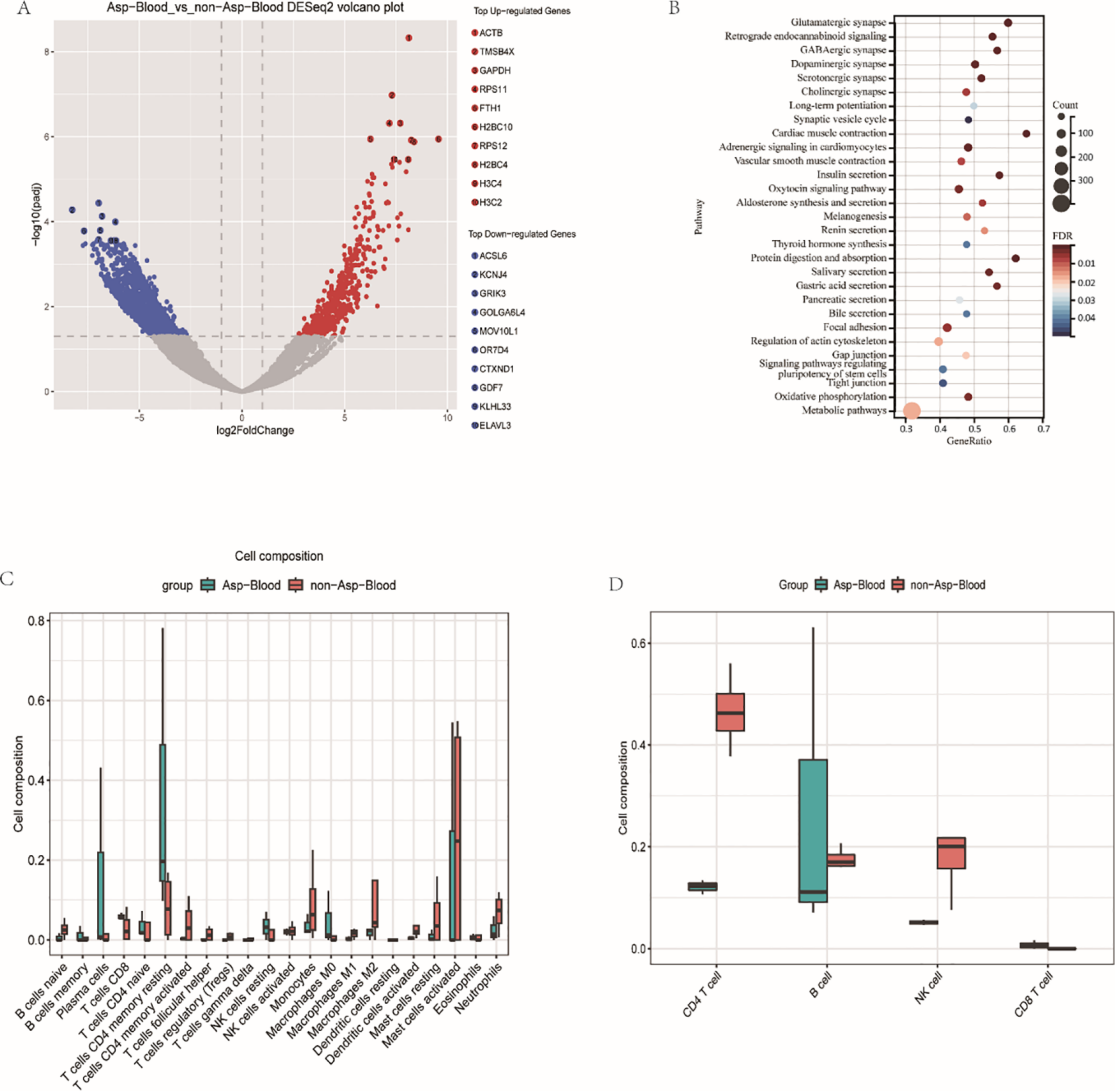
Transcriptomic and immune deconvolution analyses of blood samples from SFTS patients with IPA and without IPA. **(A)** Volcano plot of differentially expressed genes. **(B)** KEGG pathway enrichment analysis of differentially expressed genes. **(C)** Comparison of the inferred proportions of 22 immune cell types. **(D)** Composition and relative abundance of immune cells in each sample.

Between various groups of SFTS with or without IPA. we identified 4301 variably expressed genes Compared with the non-Aspergillus group (FDR ≤ 0.05 and |Log2FC|rg), the top 5 significantly upregulated genes in the IPA group included ACTB (positive regulation of cell differentiation), TMSB4X (negative regulation of inflammatory response), GAPDH (antimicrobial peptide-mediated antimicrobial humoral immune response), RPS11 (cytoplasmic translation), FTH1 (immune response). As well as ACSL6 (metabolic process), KCNJ4 (potassium ion transport), GRIK3 (G protein-coupled glutamate receptor signaling pathway), GOLGA6L4 (negative regulation of apoptosis process), MOV10L1 (germ-cell development) and other top 5 significantly down-regulated genes (**Figure 5A**). The KEGG pathways related to metabolism, cell and system function were screened. Thereinto, Insulin secretion, Gap junction and Oxidative phosphorylation were the most enriched pathways in endocrine system, cellular process and metabolism, respectively (**Figure 5B**).

The data from the sample gene expression was analyzed by deconvolution using the CIBERSORTx algorithm and the 22 immune cell marker genes provided by it, suggested that the proportion of each immune cell between the two groups was different (**Figures 5C, D**). Compared with the IPA group, the proportions of each immune cell such as CD4 T cells, B cells and dormant natural killer (NK) cells in the non-IPA group was higher, while CD8 cells were the opposite. Overall, the T cells CD4+ memory resting and Mast cells activated were in the highest abundance among immune cells. These immune cells indicated that the host has activated many cytokines to resist viral infection.

## Discussion

This retrospective study showed that patients with SFTS complicated with IPA had high morbidity and mortality. A lower absolute count of total lymphocytes and CD4+ T lymphocytes and a higher CD4/CD8 ratio were found in SFTS patients with IPA than in non-IPA patients, and the study also showed that a CD4+ T-lymphocyte count of less than 386 cells/μL may serve as a potential risk indicator for IPA in SFTS patients. Low CD4+ T-cell counts may reflect impaired cellular immunity in severe SFTS that increases susceptibility to secondary Aspergillus infection, but they may also result from immune dysregulation related to critical illness–associated immune dysfunction.

IPA is a severe opportunistic infection secondary to immunosuppression or immune impairment. It is more common in patients with immunosuppressive diseases such as granulocytopenia, patients administered high-dose corticosteroids and immunosuppressive agents, and patients with organ transplantation and acquired immunodeficiency syndrome, who have a high mortality rate (16, 17). It is well known that leukopenia is one of the critical risk factors for *Aspergillus* infection, mainly because leukopenia affects the innate and acquired immunity of patients (18). Leukocytes include neutrophils, lymphocytes, and monocytes. Macrophages are derived from monocytes. Both macrophages and neutrophils are phagocytes that play an essential role in innate immunity. Lymphocytes are important cells mediating acquired immunity and can be divided into B lymphocytes, T lymphocytes, and NK cells. B lymphocytes mainly mediate humoral immunity, while T lymphocytes mainly mediate cellular immunity. T lymphocytes are divided into helper T cells (CD4+), suppressor T cells, effector T cells, and cytotoxic T cells (CD8+) according to their functions in the immune response. During the early stage of *Aspergillus* infection, macrophages phagocytose killing spores, and inhibiting spore growth forms the first line of defense, while neutrophils phagocytose hyphae through oxidative and nonoxidative mechanisms (19). The second line of defense against *Aspergillus* is mainly cellular immunity. T cells are not only effector cells of cellular immunity but also important immune regulatory cells, among which are helper CD4+ T cells and cytotoxic CD8+ T cells, that maintain normal immune function and play an essential regulatory role in the process of cellular and humoral immunity (20, 21).

Patients with SFTS are usually physically healthy before SFTSV infection, which is associated with impaired immune function in these patients. Studies have also shown that cytokine levels are significantly increased in patients with SFTS. The levels of cytokines and chemokines expressed by severe patients are increased considerably. For example, the levels of TNF-α, IFN-γ, IL-10, IL-6, and IL-8 are significantly higher in severe patients than in mild patients (22). Many proinflammatory and anti-inflammatory mediators are produced during the acute phase, leading to systemic inflammatory response syndrome (SIRS) and compensatory anti-inflammatory response syndrome (CARS). SIRS causes cell death and organ dysfunction, and CARS causes immune suppression and increases susceptibility to secondary infections (23). The body’s antiviral immune response is the primary way to control and eliminate viral infection, and an excessive immune response leads to immunological damage. This study showed that the cellular immune function of SFTS patients was seriously impaired, especially in patients with IPA, characterized by a significant decrease in the absolute count of CD4+ T cells and a dramatic reduction in the CD4/CD8 ratio. The results of the RNA data analysis of immune cells in the study were consistent with this. Sun L et al. also reached the same results and found that the degree of CD4+ T-cell reduction was correlated with the severity of SFTS (24). Studies have also shown that the CD4+ T-cell count and Th1, Th2, and Treg cell counts are significantly reduced in nonsurvivors and are associated with the severity of SFTS. Moreover, T-cell activation, proliferation, and function are enhanced considerably, and these cells are involved in the initiation and maintenance of effective adaptive immune responses in survivors (25). Further metabolomics studies revealed that arginine deficiency in SFTS patients is greater in nonsurvivors than in survivors, which may be related to impaired T-cell function (26).

Recent studies have confirmed that IPA is a common complication of SFTS, but its pathogenesis is unclear (27, 28). The latest study showed that CD4+ and CD8+ T-cell counts are proper parameters for the early prediction of IPA in SFTS patients, with high sensitivity and specificity of cut-off values. The study confirmed that a CD4+ T-cell count of < 68 cells/mm3 combined with a CD8+ T-cell count of <111 cells/mm3 are independent risk factors for IPA in critically ill SFTS patients (28), which is similar to the results of this study.

RNA-seq was performed in our study for a better understanding of the pathogenic mechanisms of SFTSV infection. RNA-Seq as a high-throughput method for transcriptome analysis that offered more accurate measurement of expression levels of transcripts and their isoforms than other methods (29). Among the upregulated genes, previous studies have shown that Beta-actin (ACTB) has been considered as an endogenous steward gene and reference gene in cells and tissues for many years, and the expression of ACTB was associated with immune cells infiltration (30). Likewise, TMSB4X is an actin polymerization mediator, which plays a role in immune and inflammation responses, underscoring its potential role in SFTS-related pathophysiology (31).

To elucidate the changes in gene expression levels following SFTSV co-infection with Aspergillus, we conducted a KEGG pathway analysis. The results revealed that many enriched pathways belong to the endocrine system, cellular processes, and metabolism. Each pathway not only aids in our understanding of metabolic processes but also illuminates critical aspects of cellular communication and energy management. The insulin secretion pathway indicates a disruption in glucose homeostasis, which is closely related to immune responses (32, 33). Gap junctions form channels for intercellular communication, participating in inflammation, immune system activation, and responses to infection (34). Moreover, oxidative phosphorylation is a key metabolic pathway for cellular energy production, which can significantly influence the metabolism and function of immune cells (35).

Therefore, SFTSV infection affects patients’ innate and acquired immunity, making them susceptible to IPA, and the extent of immune suppression may be related to viral load. Recent studies have shown that the SFTSV load is inversely proportional to the total number of T lymphocytes and the absolute counts of CD4+ and CD8+ T cells (36). However, our study found that there was no significant difference in viral load between the IPA group and the non-IPA group. Considering that this study was a retrospective study, the measured plasma viral load at different times of onset could not reflect the real maximum viral load of patients, and whether the plasma viral load could reflect the overall viral load of the human body remains unknown.

Thrombocytopenia is also a significant cause of *Aspergillus* infection secondary to SFTS. Macrophages in the spleen can phagocytose platelets adhering to SFTSV particles, which is considered one of the leading causes of thrombocytopenia caused by SFTSV infection, and macrophages are also critical target cells of SFTSV (37). Platelets play a crucial role in inflammation and immune regulation. Platelet surfaces can express a variety of receptors and have a variety of storage particles, among which α-particles are rich in storage and contain a variety of cytokines and chemokines, which can be rapidly released at the site of inflammation or injury to regulate the activity of immune cells and induce cell migration and recruitment to the location of inflammation (38). A variety of Toll-like receptors (TLRs) and C-type lectin receptors (CLRs) can be expressed on the surface of platelets. It is speculated that the upregulation of TLR and CLR in platelets may contribute to inflammation and promote macrophage phagocytosis of fungi through their expression or stimulation (39).

Since IPA in SFTS patients is associated with a high incidence and mortality and IPA may be associated with immune suppression due to SFTSV infection, early immune booster therapy and early prophylactic antifungal therapy may be beneficial for SFTS patients. A recent study has shown that patients with SFTS who receive preventative antifungal treatment have a lower mortality rate than those who do not receive prophylactic antifungal therapy (28). However, there are no guidelines or consensus recommendations because there is insufficient medical evidence.

Our study had several limitations. First, there were few cases because of the relatively low incidence of SFTS, which may limit the precision and stability of regression estimates and is reflected by wide confidence intervals. Second, as a single center retrospective study, not all patients underwent T-lymphocyte subset testing and fungal screening, and lymphocyte subsets were not measured at standardized time points, which may have introduced selection and measurement bias. Third, the proposed CD4 cut-off and the ROC performance were not externally validated, which limits generalizability. Finally, antifungal treatment was not standardized, and variations in the timing of antifungal initiation may have influenced IPA detection and clinical outcomes.

In conclusion, this study indicated that SFTS patients had a high incidence of IPA infection, which is closely related to the impaired cellular immunity of these patients. The CD4+ T lymphocyte count decreased significantly in SFTS patients complicated with IPA. If a CD4+ T-lymphocyte count of < 386 cells/μL can identify a high risk of secondary IPA infection in SFTS patients, it can provide a specific theoretical basis for prophylactic antifungal therapy in SFTS patients.

## Data Availability

All sequence reads were deposited into the Genome Sequence Archive in the National Genomics Data Center under the accession number PRJCA055764.
