# Supplementary material for: Characteristics of T-lymphocyte subsets in patients with severe fever with thrombocytopenia syndrome complicated with invasive pulmonary aspergillosis: a retrospective study
Source: Front Immunol. 2026 Jan 23;16:1748830. doi: 10.3389/fimmu.2025.1748830 (PMC12876148; doi:10.3389/fimmu.2025.1748830)
Supplement: Supplementary file 1 [file Table1.docx]

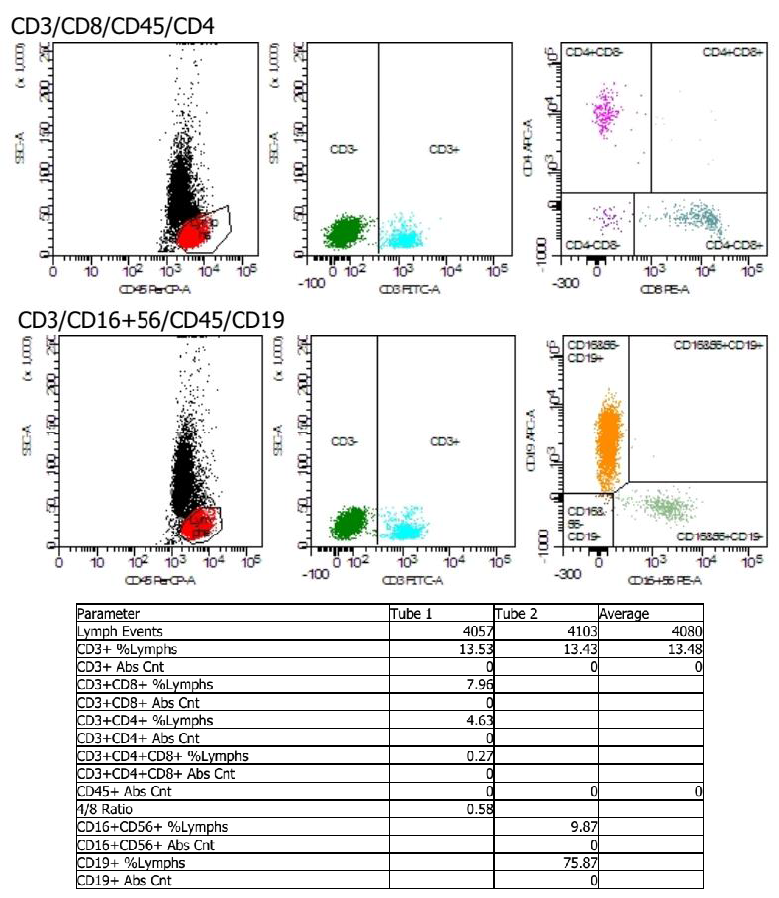


**Supplementary Figure 1** FACS plots of T-lymphocyte subsets in a 61-year-old female SFTS patient with IPA. SFTS, severe fever with thrombocytopenia syndrome; IPA, invasive pulmonary aspergillosis.


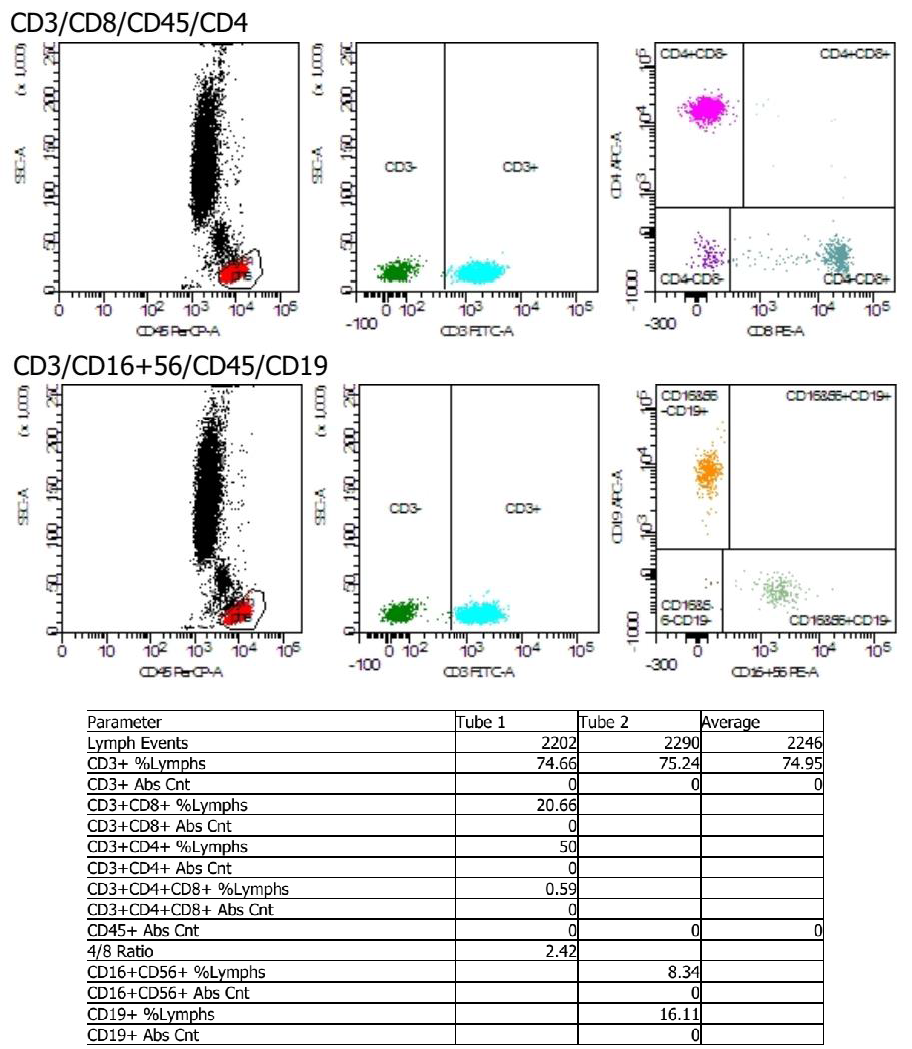
 **Supplementary Figure 2** FACS plots of T-lymphocyte subsets in a 48-year-old female SFTS patient without IPA. SFTS, severe fever with thrombocytopenia syndrome; IPA, invasive pulmonary aspergillosis.
